# Role of Oxidative Stress and Inflammation in Gestational Diabetes Mellitus

**DOI:** 10.3390/antiox12101812

**Published:** 2023-09-29

**Authors:** Renata Saucedo, Clara Ortega-Camarillo, Aldo Ferreira-Hermosillo, Mary Flor Díaz-Velázquez, Claudia Meixueiro-Calderón, Jorge Valencia-Ortega

**Affiliations:** 1 Unidad de Investigación Médica en Enfermedades Endocrinas, Hospital de Especialidades, Centro Médico Nacional Siglo XXI, Instituto Mexicano del Seguro Social, Mexico City 06720, Mexico; renata.saucedo@imss.gob.mx (R.S.); aldo.ferreira@imss.gob.mx (A.F.-H.); 2 Unidad de Investigación Médica en Bioquímica, Hospital de Especialidades, Centro Médico Nacional Siglo XXI, Instituto Mexicano del Seguro Social, Mexico City 06720, Mexico; cocamarillo2014@gmail.com; 3 Hospital de Gineco Obstetricia 3, Centro Médico Nacional La Raza, Instituto Mexicano del Seguro Social, Mexico City 02990, Mexico; mary.diaz@imss.gob.mx; 4 Departamento de Patología, Centro Médico Naval, Mexico City 04470, Mexico; sofia.meixueiro83@hotmail.com; 5 Unidad de Investigación en Reproducción Humana, Instituto Nacional de Perinatología-Facultad de Química, Universidad Nacional Autónoma de México, Mexico City 11000, Mexico

**Keywords:** oxidative stress, inflammation, gestational diabetes

## Abstract

Gestational diabetes mellitus (GDM) is one of the most common pregnancy complications. It is related to several gestational and fetal adverse outcomes. Moreover, women with GDM and their infants have a high risk of developing type 2 diabetes in the future. The pathogenesis of GDM is not completely understood; nevertheless, two factors that contribute to its development are oxidative stress and inflammation. Oxidative stress and inflammation are related; reactive oxygen species (ROS) production can activate inflammatory cells and enhance the production of inflammatory mediators. Inflammation, in turn, leads to an increased ROS release, causing a vicious circle to ensue. Inflammatory responses can be achieved via the activation of the NF-κB signaling pathway. Herein, we review the English literature regarding oxidative stress and inflammation evaluated simultaneously in the same population, attempting to identify mechanisms through which these factors contribute to the development of GDM. Furthermore, the modulation of oxidative stress and inflammation by different therapies used in women with GDM and in cell models of GDM is included in the review. Probiotics and nutrient supplementations have been shown to reduce biomarkers of inflammation and oxidative stress in vitro and in women with GDM.

## 1. Introduction

Gestational diabetes mellitus (GDM) is defined as diabetes diagnosed in the second or third trimester of pregnancy that was not overt diabetes before pregnancy [1]. It is the most common metabolic disorder of pregnant women. The worldwide prevalence of GDM ranges from <1 to 28% of all pregnancies, depending on the population studied and the diagnostic criteria used [2]. The incidence of GDM is increasing [3]. Reasons contributing to this increase include increasing maternal age, higher rates of obesity and changes in diagnostic criteria [4]. GDM is associated with adverse pregnancy outcomes and long-term maternal and offspring complications [5,6,7].

The pathophysiology of GDM is not fully understood. Women who develop GDM have a pancreatic β-cell defect that is unable to compensate for the insulin resistance of the pregnancy [8]. Factors contributing to these defects include genetic susceptibility, epigenetic changes, placental hormones, adipose tissue-derived hormones (adipokines), oxidative stress and inflammatory cytokines [9,10,11].

There is strong evidence that GDM is characterized by increased oxidative stress, an altered total antioxidant capacity (TAC) and an inflammatory profile [12,13]. Notably, these disturbances during pregnancy contribute to increased cardiovascular risk and postpartum insulin resistance [14]. Several studies have described a relationship between oxidative stress and inflammation. An increase in oxidative stress can increase the production of inflammatory cytokines, and likewise, an increase in inflammatory cytokines can stimulate the production of free radicals [15,16]. Thus, the aim of this narrative review is to examine the reported findings concerning oxidative stress and inflammation evaluated simultaneously in the same population, attempting to identify the mechanisms through which these factors contribute to the development of GDM. Furthermore, the modulation of oxidative stress and inflammation by different therapies used in women with GDM and in cell models of GDM is included in the review.

**Figure 1:**
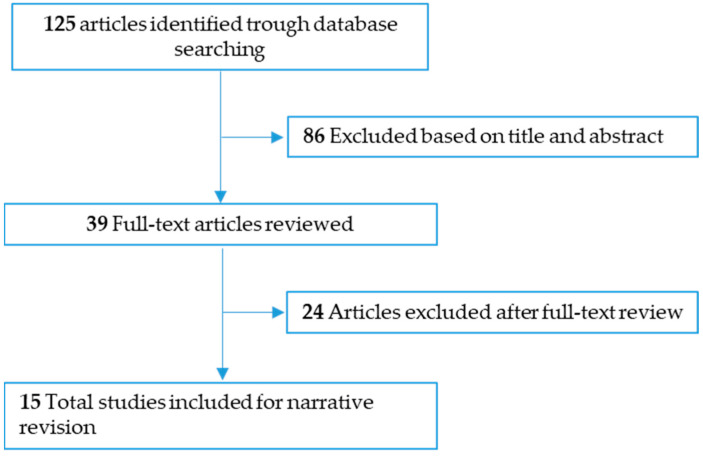
Diagram of the narrative review.

This narrative literature review was carried out from November, 2022 to August, 2023. Relevant English-language articles were reviewed through searches in PubMed and Web of Sciences using the key words “gestational diabetes”, “oxidative stress”, “inflammation” and “human”. For the description of oxidative stress and inflammation in GDM, the literature search focused primarily on observational studies that assessed oxidative stress and inflammation biomarkers simultaneously. For the description of effects of supplementation on oxidative stress and inflammation biomarkers, the literature search focused on double-blind placebo-controlled randomized clinical trials (RCTs) investigating the role of probiotics and nutrient supplementation in maternal biomarkers of oxidative stress and inflammation and in in vitro models of GDM. We excluded studies that involved animals. The search resulted in a total of 125 articles (Figure 1), which were assessed by title first and abstract second, as per our predetermined eligibility criteria. Articles whose title and abstract matched these criteria were then examined. Ultimately, 15 articles were eligible and were included.

## 2. Reactive Oxygen Species

Mitochondria are the star organelles in all cell types, as they are the energy producers and suppliers. This process provides the ATP necessary for the vital functions of the cell. However, during the process of energy production, highly reactive molecules called free radicals are also synthesized. Mitochondria also play an important role in the control of cell proliferation, as they house several proteins that trigger the intrinsic pathway of apoptosis. Thus, mitochondria have a dual role in maintaining cellular homeostasis and the body itself; any alteration in its components or functions has implications for the development of various pathologies [17].

ATP synthesis is accomplished by the formation of an electrochemical proton gradient within the inner mitochondrial membrane resulting from the biochemical reactions that make up the respiratory chain. The arrival of nicotinamide and adenine dinucleotide coenzymes (NADH) and adenine-flavine dinucleotides (FADH2), products of pyruvate metabolism, amino acids and the oxidation of fatty acids, initiates the transfer of electrons through mitochondrial complexes (I, III and IV), which will eventually be transferred to oxygen. Additionally, the output of protons through the inner mitochondrial membrane generates the mitochondrial membrane potential necessary to produce ATP. When there is a proton overload, the electron transport in complex III is partially inhibited and promotes the return of electrons to coenzyme Q and its donation to molecular oxygen, thus facilitating the production of superoxide anion (O_2_^•−^). Approximately 2% of the electrons escape the electronic transport chain and form O_2_^•−^, the main free oxygen radical or reactive oxygen species (ROS), which will give rise to other radicals of biological importance, such as hydroxyl radicals (^•^OH), peroxyl radicals (POO^•^), hydrogen peroxide (H_2_O_2_) and single oxygen (O_2_). Although mitochondria are the main organelles for the production of ROS, they can also originate in the peroxisomes, endoplasmic reticulum, cytosol and plasma membrane [18].

Hyperglycemia increases the activity of the sorbitol pathway and the accumulation of phosphate trioses (glyceraldehyde 3 phosphate and dihydroxyacetone phosphate) due to the inhibition of the glycolytic enzyme glyceraldehyde 3 phosphate dehydrogenase (G3PDH), which is capable of glycosylating proteins for the formation of advanced glycation products (AGEs), increasing protein kinase C (PKC) and activating the hexosamine pathway that generates uridine diphosphate-N-acetyl glucosamine (UDP-GlcNAc), which has the ability to bind to proteins and regulate their functions [19,20,21]. The activation of these mechanisms in addition to glucose autooxidation and mitochondrial dysfunction converges in a greater production of free radicals and oxidative stress [22].

ROS at physiological concentrations participate in various intracellular signaling pathways and thus contribute to proper cellular functioning. However, various stressors can increase ROS production and generate an oxidative stress state. In addition to mitochondrial respiration, in organisms, there are other sources of reactive species of biological importance, such as uncoupled nitric oxide synthase, peroxisomes, NADPH oxidase and the cytochrome P450 system. Reactive nitrogen species (RNS) are produced by uncoupled nitric oxide and, as their name says, contain nitrogen with different reactive capacities, such as nitric oxide (NO) and nitrogen dioxide (NO_2_). In peroxisomes, numerous oxidases, including xanthine oxidase (X/XO, for uric acid synthesis), together with cytochrome B5 and cytochrome P-450, also produce ROS [23]. NADPH oxidase is an enzymatic complex that is found in the plasma membrane. NADPH oxidase-generated ROS have an important role in cell signaling, the innate immune response and cell proliferation, including oncogenic transformation [24]. Therefore, cells have mechanisms that limit and/or neutralize the production of ROS.

The body’s antioxidant defenses are classified into enzymatic and nonenzymatic defenses. The first group is composed of the enzymes superoxide dismutase (SOD) with three isoforms (SOD1, dependent on CuZn; SOD2, dependent upon Mn and SOD3, extracellular), catalase and glutathione peroxidase (GPx). SOD catalyzes the dismutation of O_2_^•−^ into H_2_O_2_, which is transformed into water and oxygen by the action of catalase and GPx and thus prevents it from being integrated into the Fenton reaction and the production of ^•^OH. This radical is one of the most reactive, as it quickly interacts with lipids, proteins and DNA, causing oxidative damage. The second group includes vitamins C and E, bilirubin, biliverdin, uric acid, glutathione (GSH) and flavonoids, of which GSH is the most abundant intracellular antioxidant [25,26].

### 2.1. Oxidative Stress

The imbalance between ROS production and the activation of antioxidant systems leads to a state known as “oxidative stress”. This highlights the production of ROS and the oxidation of macromolecules that result in cellular and tissue damage. It is known that the body’s antioxidant defenses can decrease and/or be lost under certain circumstances, resulting in increased ROS and oxidative stress. Uncontrolled production of ROS alters metabolism and can induce cell death. ROS quickly react with biomolecules (DNA, proteins, lipids and carbohydrates), oxidize them and cause irreparable damage, disrupt homeostasis and lead to cell loss.

The increase in the superoxide anion in the respiratory chain is an event that triggers the activation of glucotoxicity mechanisms.

The •OH radical attacks DNA and generates important mutations and alterations at the transcriptional level. Oxidative damage to DNA can be measured by the presence of 8-hydroxy-2’-deoxyguanosine (8-OHdG). DNA attack by RNS causes purine nitration. Proteins experience oxidation and nitration in their amino acids by the action of ROS and RNS, with a subsequent loss of amino acids and biological functions. Oxidative damage can be evaluated by the concentration of carbonyl groups and tyrosine nitration. Glycosylation refers to the interaction between the lysine and arginine residues of proteins with glucose to form early-stage Amadori products or fructosamine. Through molecular rearrangements and oxidations, α-dicarbonyl compounds (α-oxoaldehydes), such as 3-deoxyglucosone, methylglyoxal (MGO) and glyoxal, are precursors of AGEs. These compounds can simultaneously combine with two reactive groups of proteins, favoring their aggregation and decreasing biological activity [27]. Glycosylation is another modification that proteins such as albumin, hemoglobin or low-density lipoproteins (LDL) can undergo when the concentration of glucose increases in the body.

Oxidative stress has been identified as part of the physiopathology of various diseases, among which metabolic diseases (metabolic syndrome and diabetes) are highlighted, although it is also identified in obesity and gestation. In this context, increased glucose and lipids, which often accompany metabolic diseases, promote ROS production. Due to the increased electron supply in the mitochondrial respiratory chain, increased NADH and FADH2 and the activation of other metabolic pathways also produce ROS.

Increased ROS promote insulin secretion alterations, pancreatic β-cell damage and death [28]. Mitochondrial membrane potential alterations due to ROS increase decreased ATP production and glucose-induced insulin secretion [29]. In addition, mitochondrial permeability changes induce proapoptotic protein (cytochrome c, apoptosis-inducing factor, among others) release and β-cell apoptosis [30]. It is known that β-cells have low antioxidant protection, and ROS damage is too severe [31]. Therefore, when oxidative stress increases, pancreatic β-cell apoptosis and loss frequently occur.

### 2.2. Oxidative Stress and Its Interaction with Inflammation

Inflammation is a series of molecular and cellular responses that protect the body from infections and other insults. The inflammatory response is maintained until the stimulus is controlled or disappears and is known as acute inflammation; if this does not occur, then there are indications of chronic inflammation [32]. The inflammatory response involves the main cells of the immune system (neutrophils, basophils, mast cells, T cells, B cells, etc.). However, the presence of specific leukocytes in certain lesions has been demonstrated. The need for the strict regulation of cytokines, growth factors, eicosanoids (prostaglandin, leukotrienes, etc.), complement and peptides, as well as of the intracellular signaling needed, will depend on the type of instigator or aggressor (pathogenic, molecules) and/or involved tissues [33].

Alterations in metabolism can also induce chronic inflammation due to the presence of some endogenous molecules, such as AGEs, oxidized lipoproteins, saturated free fatty acids (palmitic), cytokines and hyperglycemia, in addition to molecules secreted by adipose tissue. This type of inflammation has been associated with the development of pathophysiological processes linked to a greater production of free radicals and oxidative stress [34], events present in metabolic diseases such as obesity, metabolic syndrome, type 2 diabetes (T2D) and GDM. Fatty free acids and AGEs are recognized by Toll-like receptors (TLRs activated principally by molecules associated with pathogenicity and damage), which lead to the activation of nuclear factor kappa B (NF-κB) and to the activation of the transcription of inflammatory cytokines such as TNF-α (factor of necrosis tumor alpha), IL-1β and CXCL8 or IL-8 (interleukins) [35].

In recent years, it has been shown that the accumulation of adipose tissue in the body triggers a chronic inflammatory response of a low degree. This is identified by the arrival of macrophages and T lymphocytes to adipose tissue and the increase in proinflammatory cytokines such as TNF-α, IL-1, IL-6, IL-17 and reactive C protein (PCR) [36]. The accumulation of fatty acids in adipocytes promotes the production of free radicals and lipoperoxidation, which can induce apoptosis in these cells. In addition, necrosis of adipocytic cells has been demonstrated due to a lack of blood irrigation and oxygen in some regions of visceral adipose tissue. This attracts phagocytes for the removal of dead cells and their waste, thus initiating the local inflammatory process. This increases the levels of IL-1β, IL-6, TNF-α and leptin, as well as the secretion of chemoattracting factors (monocyte chemoattractant protein–1) that stimulate macrophage arrival and exacerbate the inflammatory environment.

The inflammation of adipose tissue can spread to pancreatic islets and other tissues, mainly in an insulin-dependent manner. This is evidenced by the presence of high levels of inflammatory markers in the serum of obese patients with insulin resistance [37]. TNF-α levels correlate with the body mass index and promote IL-6 expression, exacerbating hepatic triacylglycerol secretion and hypertriglyceridemia [38].

In pancreatic β cells, increases in non-esterified fatty acids, particularly saturated fatty acids, are harmful, among other pathways, through the induction of IL-β, IL-6 and IL-8 [36,39], which affect the synthesis and secretion of insulin [40]. Obese patients frequently have glucose metabolism and insulin response alterations. This constitutes a constant stimulus on β cells, which increases the synthesis and accumulation of misfolded proteins, including insulin, for which homeostasis is lost and endoplasmic reticulum (ER) stress is generated, along with oxidative stress, increased inflammation and apoptotic pathways and glucose intolerance [36,40]. The increase in ROS also activates several inflammatory cascades, which lead to the activation of NF-kB, monocyte chemotactic protein 1, nitric oxide, TGF-β and IL-1β, thus contributing to the exacerbation of the inflammatory state [40].

## 3. Relationship between Oxidative Stress and GDM

Physiological pregnancy increases oxidative stress, resulting in high levels of circulating ROS. The main source of ROS during pregnancy is the placenta. Increased oxidative stress is counterbalanced by increased antioxidant synthesis [41].

Placentas from women with GDM show a higher release of 8-isoprostane, a marker of lipid peroxidation, compared to placentas from normal pregnancies. Likewise, the GDM placenta also increases the expression of xanthine oxidase (XO), malondialdehyde (MDA), 4-hydroxynonenal (4-HNE) and protein carbonyl. When oxidative stress surpasses the antioxidant defense in the placenta, oxidative damage can spread to distal tissues. GDM is characterized by increased maternal circulating levels of oxidative stress markers (higher than in physiological pregnancy) and an altered antioxidant defense. The reactive oxygen species XO, MDA, thiobarbituric acid reactive substances (TBARS) and lipid hydroperoxide (LOOH) are significantly increased, and TAC is significantly decreased in GDM [12].

Of note, hyperglycemia induces oxidative stress, and the majority of investigations have been conducted after the diagnosis of GDM, in the second or third trimester. There are few studies evaluating the potential association between oxidative stress markers in early pregnancy and the subsequent risk of GDM [42]. Qiu et al. (2011), in a pilot nested case‒control study, identified elevated maternal urinary 8-OHdG, a sensitive marker of oxidative DNA damage and repair produced by the oxidation of the nucleoside deoxyguanosine, in early pregnancy, and it was associated with GDM risk [43]. In addition, in a recent case–control study based on the study cohort of a Hungarian biobank, Gerszi et al. (2023) found that serum TAC was significantly and independently increased at the end of the first trimester in women who developed GDM later in pregnancy, suggesting an elevated level of free radicals that is compensated by an increased production of endogenous antioxidants [44]. On the basis of these findings, several studies have evaluated the role of a plant-based diet (rich in antioxidants, fibers, magnesium and potassium and low in saturated/trans fats) before pregnancy and in early pregnancy in the development of GDM. Importantly, the literature suggests that this kind of diet may decrease the incidence of GDM, and it also prevents T2D [21,45].

The increase in ROS and oxidative stress leads to impaired insulin secretion and insulin resistance [46]. Pancreatic β-cells are particularly sensitive to ROS because they have low levels of free radical quenching antioxidant enzymes such as catalase, glutathione peroxidase and superoxide dismutase [47]. Therefore, oxidative stress induces β-cell dysfunction via the induction of apoptotic events, impairing KATP channels and inhibiting transcription factors involved in β-cell neogenesis such as Pdx-1 and MafA and mitochondrial dysfunction, leading to decreased insulin production [48]. On the other hand, oxidative stress can impair insulin signaling, leading to lower insulin sensitivity in peripheral tissues. In vitro, ROS and oxidative stress lead to the activation of multiple serine kinase cascades. These activated kinases decrease the insulin-stimulated tyrosine phosphorylation of the insulin receptor (IR) and the family of IR substrate proteins, attenuating insulin action [49]. Additionally, oxidative stress can reduce GLUT-4 expression and content in adipose tissue and skeletal muscle, reducing the cellular uptake of glucose and thereby inducing insulin resistance [50]. Oxidative stress can also disturb insulin production and insulin signal transduction via the promotion of inflammatory responses.

## 4. Relationship between Inflammation and GDM

Circulating inflammatory cells, such as monocytes, neutrophils and proinflammatory cytokines (e.g., IL-1β, IL-6 and TNF-α), are upregulated in GDM. Cytokines are produced by cells of the immune system, placenta and adipose tissue [13]. As the placenta and adiposity increase during pregnancy, there is an enhanced secretion of proinflammatory cytokines. Additionally, the activation of inflammatory pathways also occurs as a response to elevated glucose concentrations. The pro-inflammatory mediators induce the production of other pro-inflammatory cytokines and chemokines (CXCL1, CXCL5 CXCL8, CCL2). Proinflammatory cytokines inhibit insulin release from β-cells and impair insulin signaling, inducing Janus kinase pathways (JNKs), which in turn stimulate IRS-1 serine phosphorylation, leading to an impairment in insulin action [51]. Higher levels of TNF-α and high-sensitivity CRP (hs-CRP), an acute-phase inflammatory protein, have been studied as markers for GDM. Prospective studies have shown that these inflammatory factors measured at 11–14 weeks of gestation are predictive of GDM [52,53].

## 5. Studies Evaluating the Relationship between Oxidative Stress and Inflammation in GDM

Numerous studies have assessed oxidative stress and inflammatory biomarkers in GDM in an independent way. However, to the best of our knowledge, few studies have assessed these biomarkers simultaneously. To date, ten human studies have been published describing oxidative stress and inflammation in GDM women from different ethnic groups. Their characteristics are displayed in Supplementary Table S1. The studies showed heterogeneity regarding the sample size, diagnostic criteria, gestational age at sampling, type of sample and oxidative stress and inflammation markers. Most of the studies have used maternal peripheral blood samples, either serum or plasma. Others have assessed insulin-resistant peripheral maternal tissues, such as subcutaneous (SAT) and visceral adipose tissue (VAT) and skeletal muscle. Furthermore, the placenta was also analyzed. Some tissue studies have evaluated oxidative stress and inflammation markers under basal conditions and others under oxidative stress exposure. Finally, there is diversity in the methods for the quantification of biomarkers, and the majority of the studies have used colorimetric methods for oxidative stress and immunoassays for inflammation. However, proteomic studies using sensitive techniques such as liquid chromatography–mass spectrometry (LC–MS) have been conducted recently to construct metabolic profiles and identify novel pathways in GDM [54,55,56,57,58,59,60,61,62,63].

### 5.1. Studies in Maternal Serum Or Plasma

During the last 10 years, oxidative stress and inflammatory biomarkers have been detected simultaneously in the blood of women with GDM, primarily during the second and third trimesters. Notably, no difference in the total oxidant status between GDM and controls has been reported. However, the levels of MGO, an oxidizing substance, are significantly higher in GDM women than in normal pregnant women. In addition, the total antioxidant status and the enzymatic antioxidants GPX and SOD are decreased in women with GDM when compared to controls. On the other hand, women with GDM show increased levels of TNF-α, IL-8 and CRP at diagnosis [33,34,35,37]. TNF-α and CRP have been found to be elevated in the first trimester of women who subsequently develop GDM, suggesting a potential role in glucose metabolism regulation and a potential value as diagnostic biomarkers [52,53].

The interplay between oxidative stress and inflammation in the peripheral blood of GDM patients has been reported. Recent proteome studies have shown protein coregulation between oxidative stress and inflammation in GDM; in particular, Liu et al. (2020) identified protein coregulations among 52 inflammatory system proteins and 4 antioxidant proteins (extracellular superoxide dismutase, peroxiredoxin-1, serum haptoglobin and paraoxonase/arylesterase 1) [62]. In addition, Kopylov et al. (2020), analyzing a proteomoc map, postulated that ROS generation stimulates NF-kB signaling and TNF-α and IL-6 production in GDM [63].

Oxidative stress and inflammation biomarkers are also associated with metabolic features of GDM. Piuri et al. (2020) showed that PAF and MGO were positively associated with HOMA-IR and HbA1c at diagnosis [61]. Furthermore, the study by Ozler et al. (2019) illustrated that increased TNF-α levels and decreased TAS levels were independent predictors of the need for insulin treatment in GDM patients [57]. These results suggest that high glucose levels modulate oxidative stress and inflammatory marker levels. Remarkably, in addition to the association of MGO with glucose metabolism, MGO has also been positively correlated with both pre-pregnancy weight and GDM diagnosis [61]. In this regard, evidence suggests that maternal obesity is associated with greater oxidative stress [64].

### 5.2. Studies in Placenta

Different approaches have been used to evaluate markers of oxidative stress and inflammation in placentas from women with GDM at term. Their mRNA expression and release under basal conditions and in response to oxidative stress have been measured. Under basal conditions, the release of 8-isoprostane, MDA and the antioxidant gene expression from catalase and glutathione reductase are higher in placental samples from GDM women compared to controls. In contrast, the release of TNF-α, IL-6 and IL-8 was not different between the two groups in most studies. Only one study showed higher IL-6 levels in GDM [60]. Furthermore, in response to oxidative stress by X/XO, LPS and HX/XO, the placenta did not show changes in 8-isoprostane release or antioxidant enzyme expression but showed a higher release of inflammatory markers. However, this last response was blunted compared to the control group [54,55,56,60]. These data suggest that the GDM placenta has a reduced capacity to respond to oxidative stress. Lappas et al. (2010) hypothesized that the GDM placenta may be preconditioned by transient intracellular oxidative stress, which attenuates its responsiveness to further oxidative insult [56].

In addition, the study by Li et al. (2019) provided further evidence of the link between oxidative stress and inflammation in GDM [60]. This study demonstrated a positive association between MDA and IL-6 and a negative association between MDA and adiponectin, an anti-inflammatory adipokine that improves insulin sensitivity and is often decreased in the presence of GDM [60].

### 5.3. Studies in SAT and VAT Adipose Tissue

Under basal conditions, 8-isoprostane release is greater in GDM SAT, and stimulation with LPS and HX/XO increases its release in SAT and VAT as well as TNF-α, IL-6 and IL-8 release, while there is no change in SAT and VAT antioxidant gene expression under basal conditions and in response to HX/XO [55,56].

### 5.4. Studies in Skeletal Muscle

Interestingly, a study investigated markers of oxidative stress and inflammation in skeletal muscle obtained from women with GDM. Under basal conditions, 8-isoprostane release was greater in GDM, and stimulation with LPS increased its release. On the other hand, there was no difference in the release of TNF-α, IL-6 and IL-8 under basal conditions, but stimulation with LPS resulted in greater release of IL-6 and IL-8 [55].

**Figure 2:**
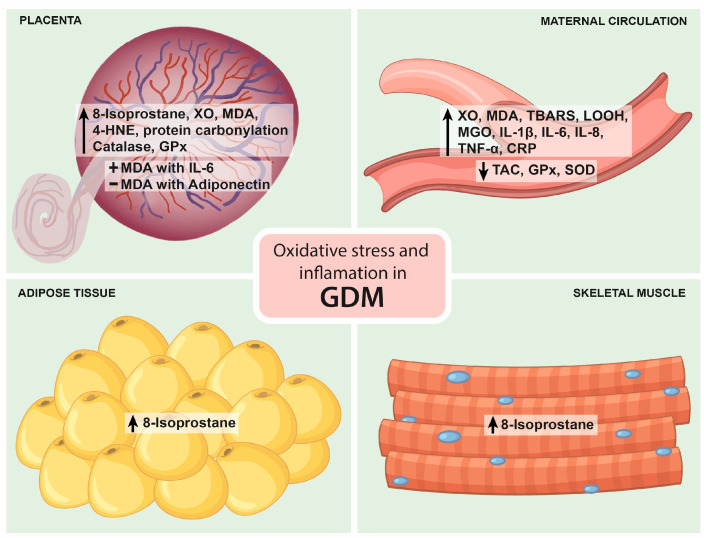
Main findings on oxidative stress and inflammation in GDM. The figure depicts the major findings regarding oxidative stress and inflammation in GDM in the placenta, maternal circulation, adipose tissue, and skeletal muscle. Arrows represent increased or decreased concentrations or upregulation or downregulation, + indicates a positive correlation, and − indicates a negative correlation. Further details are provided in the text. XO: xanthine oxidase; MDA: malondialdehyde; 4-HNE: 4-hydroxynonenal; GPx: glutathione peroxidase; IL: interleukin; TBARS: thiobarbituric acid reactive substances; LOOH: lipid hydroperoxide; MGO: methylglyoxal; TNF-α: tumor necrosis factor alpha; CRP: C-reactive protein; TAC: total antioxidant capacity; SOD: superoxide dismutase; GDM: gestational diabetes mellitus.

Figure 2 summarizes the main findings on oxidative stress and inflammation in GDM.

The association between oxidative stress and inflammation biomarkers in GDM may vary in different populations by the kind of diet and by some other factors such as physical activity, maternal age, obesity, smoking and the pharmacological management of GDM. Interestingly, consistent evidence shows that GDM exhibits increased markers of oxidative stress and inflammation and altered antioxidant defenses in circulation, placenta, adipose tissue and skeletal muscle when compared to normal glucose-tolerant pregnancies. However, the results obtained from these studies may not represent the global population, as a majority were conducted in Australia and China. Furthermore, the main limitation of the studies is the relatively small size of the groups. Therefore, future studies in GDM women from different geographical locations and with larger sample sizes are needed to confirm the findings.

## 6. Effect of Supplementation on Oxidative Stress and Inflammation Biomarkers

### 6.1. In Women with GDM

The primary approach in GDM management is a healthy lifestyle recommendation with diet and exercise. This lifestyle management has been shown to reduce macrosomia, shoulder dystocia, and preeclampsia. However, studies have reported that 13% of women do not achieve glycemic targets (fasting glucose < 95 mg/dL) with lifestyle management and require supplementary hypoglycemic agents (i.e., insulin and oral pharmacotherapy such as metformin and glyburide) [65]. Insulin is the first-line therapy, and due to its large molecular size, it does not cross the placenta. Oral pharmacotherapy is associated with improved cost effectiveness, compliance and acceptability compared to insulin therapy [66]. However, unlike insulin, metformin and glyburide cross the placenta, and the long-term safety for the offspring of women with GDM is still uncertain. On the other hand, treatment failure with metformin is 33.8%, and treatment failure with glyburide is 20%; therefore, women require the addition of insulin to maintain adequate glycemic control [67,68]. All these therapeutics attempt only to manage the resulting hyperglycemia and have no effect on the inflammation and oxidative stress associated with GDM. Therefore, an additional novel management strategy with therapeutics such as probiotics and nutrient supplementation has been reported (Supplementary Table S2).

Recent RCTs have investigated the effect of probiotic supplements and nutrient supplementation, including magnesium, zinc, calcium and their coadministration with vitamin D, on inflammation and oxidative stress biomarkers in GDM. Several studies have reported a link between altered gut microbiota and GDM [69], and due to this interaction, Hajifaraji M et al. (2018) investigated the effects of probiotics containing four bacterial strains, Lactobacillus acidophilus LA-5, Bifidobacterium BB-12, Streptococcus thermophilus STY-31 and Lactobacillus delbrueckii bulgaricus LBY-27, on inflammation and oxidative stress markers in women with newly diagnosed GDM. They found that probiotic supplementation for 8 weeks decreased serum hs-CRP, TNF-α, erythrocyte GPx and MDA and increased glutathione reductase levels [70]. Evidence suggests that probiotics exert biological effects through the inhibition of the NF-κB pathway, which decreases oxidative stress [71].

However, multinutrient therapy has shown positive effects on inflammation and oxidative stress in women with GDM. Jamilian et al. (2019), in a randomized, double-blind, placebo-controlled trial among women with GDM who were not on oral hypoglycemic agents, showed that co-supplementation with magnesium–zinc–calcium–vitamin D for 6 weeks significantly reduced serum hs-CRP and plasma MDA concentrations and increased TAC levels compared to the placebo [72].

Interestingly, the newborn weight and the rate of macrosomia were lower in the magnesium–zinc–calcium–vitamin D group than in the placebo group. These effects might be explained by the role of magnesium, vitamin D and zinc in regulating NF-κB activity and ROS production [73,74].

All these studies were carried out for the treatment of GDM, and the results in all of them were positive, showing a decline in inflammation and oxidative stress markers, suggesting their potential therapeutic use in the treatment of the disease. However, the main limitations of the studies were the short duration of the interventions, the lack of a control group of healthy pregnant women and the failure to investigate the effect of supplementations on pregnancy and fetal and long-term offspring outcomes. Thus, future research with a longer duration of the interventions on maternal and fetal health is needed. It is worth mentioning that screening and treatment for GDM occur at 24 weeks of gestation, and therefore, studies about supplementation for the prevention of GDM are necessary.

### 6.2. In Vitro Models of GDM

Additionally, in vitro models of GDM have also been used to establish the effect of phenolic acids–natural compounds found in many common fruits and vegetables, such as pomegranates and curcumin, on inflammation and oxidative stress. The findings of these studies are presented in Supplementary Table S2. Nguyen-Ngo C 2019 et al. (2019) determined the effect of naringenin, a polyphenol with antidiabetic, anti-inflammatory and antioxidative properties, on glucose metabolism, inflammation and oxidative stress in an in vitro human tissue explant model of GDM [75]. To develop a GDM-like environment, the authors stimulated human placenta, VAT and skeletal muscle from normal glucose-tolerant women at term with elective Cesarean sections and with TNF-α, and, to determine the effects of naringenin, the tissues were incubated with or without 400 µm naringenin. Naringenin treatment significantly improved TNF-α-impaired glucose uptake in skeletal muscle. In the placenta and VAT, naringenin significantly reduced the expression of pro-inflammatory cytokines and chemokines and increased antioxidant mRNA expression. Moreover, in the placenta, naringenin suppresses NF-κB [75].

Another recent study by Nguyen-Ngo C et al. (2020), using an in vitro human tissue explant model of GDM, found that punicalagin and curcumin significantly reduced proinflammatory cytokine and chemokine mRNA expression and secretion and increased anti-inflammatory cytokine mRNA expression in the human placenta, VAT and SAT [76]. Interestingly, punicalagin exerted a potent anti-inflammatory effect in the human placenta, VAT and SAT. On the other hand, curcumin exerted a more effective anti-inflammatory response in the placenta. The authors also reported that curcumin significantly increased catalase mRNA expression in the placenta, while punicalagin reversed TNF-α -induced increases in SOD2 mRNA expression in the placenta, VAT and SAT. Curcumin was also found to reduce the H_2_O_2_ concentration in VAT tissue lysate. These findings suggest that punicalagin and curcumin exert tissue-dependent effects [76].

Notably, low maternal levels of selenium, an antioxidant essential trace element naturally present in many foods, are associated with an increased risk of developing GDM, and selenium supplementation in pregnant women with GDM improves glucose homeostasis [77,78]. For this reason, a study by Nguyen-Ngo C et al. (2022) assessed the effect of selenium using the in vitro human tissue explant model of GDM described above [79]. They found that selenium pretreatment blocked LPS- and TNF-α-induced mRNA expression and the secretion of proinflammatory cytokines and chemokines while increasing anti-inflammatory cytokine and antioxidant mRNA expression in the placenta, VAT and SAT. Selenium pretreatment was also found to inhibit the LPS- and TNF-α-induced phosphorylation of ERK in the placenta, VAT and SAT [79]. A study performed by Ruiz-Palacios showed that the ERK signaling pathway is increased in placentas from women with GDM compared to healthy pregnant women [80]. On the other hand, Lappas et al. (2007) showed that ERK inhibition prevented the inflammation-induced expression of pro-inflammatory cytokines and chemokines in the placenta [80]. Selenium is also known to suppress the NF-κB pathway [81]. These findings indicate that selenium may be able to prevent the inflammation and oxidative stress associated with GDM.

The principal limitation of the above-mentioned studies is the model of GDM. It uses TNF-α to induce inflammation and is considered a general model of inflammation. Furthermore, the authors studied tissues obtained at term; thus, they only represent the effect of phenolic acids and selenium at a specific time in the pregnancy. Further studies with other in vivo models of GDM and at different stages of pregnancy are necessary to identify the effect of phenolic acids and selenium on antioxidant and anti-inflammatory activity.

## 7. Conclusions

Consistent evidence shows that GDM exhibits increased markers of oxidative stress and inflammation and altered antioxidant defenses in circulation, placenta, adipose tissue and skeletal muscle when compared to normal glucose-tolerant pregnancies. In addition, the findings suggest that ROS activate the transcription factor NF-kB, which induces the transcription of several inflammatory mediators. Some biomarkers of oxidative stress and inflammation are elevated starting in the first trimester in women who subsequently develop GDM. This provides the opportunity to predict the development of GDM before glucose intolerance is detectable and to promote early interventions intended to prevent the development of maternal and fetal complications and to reduce their associated healthcare costs.

Importantly, therapeutic approaches such as probiotic and nutrient supplementation have been shown to reduce biomarkers of inflammation and oxidative stress in vitro and in women with GDM. However, the clinical significance of this decrement and its potential adverse effects on the mother-fetus binomial compared to those of pharmacological interventions are unknown. Therefore, more evidence of the effects of this kind of supplementation on maternal and fetal health is still needed.

## Data Availability

Not applicable.

## Supplementary Materials

The following supporting information can be downloaded at: https://www.mdpi.com/article/10.3390/antiox12101812/s1, Table S1: Studies assessing the role of oxidative stress and inflammation in gestational diabetes, Table S2: Effect of supplements on oxidative stress and inflammation in women with GDM and in cell models of GDM.
