# Supplementary material for: Role of Oxidative Stress and Inflammation in Gestational Diabetes Mellitus
Source: Antioxidants (Basel). 2023 Sep 29;12(10):1812. doi: 10.3390/antiox12101812 (PMC10604289; doi:10.3390/antiox12101812)
Supplement: Supplementary file 1 [file antioxidants-12-01812-s001.zip › antioxidants-2609947-supplementary.pdf]

antioxidants MDPI

Supplementary Table S1. Studies assessing the role of oxidative stress and inflammation in gestational diabetes.

Author, Year [Reference]	Country	Study design	Sample size	Diagnostic criteria	Age	BMI	GA at sampling	Sample	Oxidative stress biomarker	Inflammatory biomarker	Main findings
Coughlan, M.T., 2004 [54]	Australia	Case–control study	GDM: 16 CON: 16	Australasian Diabetes in Pregnancy Society, fasting venous plasma glucose level of 5.5 mmol/L or more (99 mg/dl) glucose, and/or 8.0 mmol/L or more (144 mg/dl) glucose 2 h after a 75-g oral glucose load at approximately 28 wk gestation	GDM: 31.5±6.1 CON: 33.3±4.0	GDM: 30.3±9.9 CON: 24.4±3.9	GDM 38.5±0.6 CON 39.0±0.6	Placental tissue incubated in the absence and presence of X/XO system.	8-isoprostane measured by a competitive ELISA	IL-6, IL-8, TNF-α, measured by a sandwich ELISA. NF-kB DNA-binding activity determined by EMSA	No differences were observed in the release of TNF-α, IL-6, and IL-8 from CON or GDM placentae under basal condition. However, 8-isoprostane release was 2-fold greater in the GDM group. In response to X/XO, TNF-α and 8-isoprostane release and NF-kB DNA binding activity were significantly increased in CON (20-fold, 2-fold, and 35%, respectively). In contrast, the response of GDM tissues to oxidant stress was blunted, with no change in 8-isoprostane release, a 4-fold increase in TNF-α release, and a 40% reduction in NF-kB DNA binding activity.
Lappas, M., 2004 [55]	Australia	Case–control study	GDM: 12 CON: 10	Australasian Diabetes in Pregnancy Society	GDM: 35.3±1.6 CON: 31.1±1.0	GDM: 25.7±1.6 CON: 28.5±2.0	GDM: 38.8±0.2 CON: 38.7±0.2	Placenta, subcutaneous adipose tissue and skeletal muscle, incubated in the absence and presence of LPS, TNF-α, IL-6 or IL-8.	8-isoprostane measured by a competitive ELISA	IL-6, IL-8, TNF-α, measured by a sandwich ELISA	In all three tissues, 8-isoprostane release was greater in women with GDM, and stimulation with LPS increased 8-isoprostane release from adipose and skeletal muscle, but not placenta, obtained from women with GDM. In tissues obtained from CON women, LPS stimulation increased 8-isoprostane release in placenta and had no effect on adipose tissue and skeletal muscle. There was no difference in the release of TNF-α, IL-6, and IL-8 from placenta, adipose tissue, and skeletal muscle obtained from CON and GDM. Stimulation of placenta, adipose tissue, and skeletal muscle with LPS and TNF-α resulted in greater release of IL-6 and IL-8, whereas only LPS increased TNF-α release from all three tissues.
Lappas, M., 2010 [56]	Australia	Case–control study	GDM: 17 CON: 23	Australasian Diabetes in Pregnancy Society	GDM: 32.6±1.0 CON: 31.8±0.8	GDM: 30.0±2.0 CON: 28.9±1.5	GDM: 38.2±0.2 CON: 38.8±0.1	Placenta, subcutaneous and omental adipose tissue, incubated in the absence and presence of HX/XO	CAT, GSR, GPX, and SOD gene expression was determined by RT–PCR. 8-isoprostane release was measured by a competitive ELISA.	The release of cytokines (IL1β, IL2, IL4, IL5, IL6, IL7, IL8, IL10, IL12, IL13, IL17, G-CSF, GM-CSF, IFNγ, MCP1, MIP1β, TNF- α) in placenta was performed using the Bio-Plex suspension assay system and 17-plex human cytokine assay kit.	CAT and GSR mRNA expression was higher in GDM compared with CON placenta. There was no difference in GPX and SOD mRNA expression. Antioxidant gene expression was unaltered between CON and GDM adipose tissue. HX/XO treatment significantly stimulated cytokine release (13/16 cytokines) and cytokine mRNA expression, and decreased

										IL-6, IL-8, TNF- α release in adipose tissue was measured by sandwich ELISA, and gene expression by RT–PCR.	antioxidant gene expression (CAT and GSR) in placenta from CON. In GDM placenta, HX/XO only significantly increased the release of 3/16 cytokines, while there was no effect on antioxidant gene expression. In CON and GDM adipose tissues, HX/XO increased proinflammatory cytokine and 8-isoprostane release, while there was no change in antioxidant gene expression.
Ozler, S., 2019 [57]	Turkey	Prospective case–control study	GDM: 58 CON: 30	IADPSG criteria, 75 g oral glucose tolerance test (OGTT), GDM is diagnosed with abnormal glucose level in any of the 3 glucose values (plasma glucose thresholds: fasting 92 mg/dL, 1-h 180 mg/dL and 2-h 153 mg/dL)	GDM: 29.4±3.7 CON: 29.9±3.2	GDM: 29.5±4.5 CON: 30.8±3.6	GDM: 24-28 CON: 24-28	Serum	TOS, TAS, PON, SPON, and ARES determined spectrophotometrically. OSI calculated according to the formula: OSI [arbitrary unit(AU)]= TOS (μmol H2O2 eqv./L)/TAS (mmol Trolox eqv./L)× 1002.	TNF-α measured by ELISA	TNF-α and TAS were found to be predictive for GDM at 24–28 weeks of gestation. In addition, increased TNF-α and lower TAS levels were independent predictors of the need for insulin treatment in GDM patients.
Zhu, C., 2015 [58]	China	Case–control study	GDM: 36 CON: 36	IADPSG criteria	GDM: 31.2±4.0 CON: 30.4±3.7	GDM: 21.2±1.9 CON: 20.5±2.2	GDM: 24-28 CON: 24-28	Plasma	CER and Trf were measured by enzyme-linked immunonephelometry. 3-NT was identified by a double-antibody sandwich ELISA	hs-CRP measured by enzyme-linked immunonephelometry	hs-CRP levels were higher in pregnant women with GDM compared to CON subjects both at fasting and at 1-h OGTT. CER, Trf, and 3-NT increased after the OGTT but no significant differences were found between the GDM and CON groups.
Sudharshana Murthy, K.A., 2018 [59]	India	Prospective case–control study	GDM: 30 CON: 30	Diabetes In Pregnancy Study group, India criteria, 75 g glucose, and 2 h later, plasma glucose of 140 mg/dl or more was diagnostic of GDM	GDM: 27.6 CON: 23.3	GDM: 25.7 CON: 25.0	GDM: 24-28 CON: 24-28	Serum	GTX, SOD estimated by calorimetric method	TNF-α, IL-6, IL-8 evaluated by ELISA	TNF-α and IL-8 levels were significantly increased and GTX and SOD were significantly reduced in GDM group. TNF-α levels were associated with preeclampsia in GDM women.
Li, H., 2019 [60]	China	Case–control study	GDM: 72 CON: 80	IADPSG criteria	GDM: 28.8±4.6 CON: 27.9±5.8	GDM: 23.2±3.2 CON: 22.9±2.7	GDM: 38.8±1.2 CON: 39.1±1.0	Placental tissue lysate	MDA measured by thiobarbituric acid method. AGEs measured by ELISA	IL-6 and adiponectin measured by ELISA	The levels of MDA, AGEs and IL-6 were significantly higher in GDM than CON group. Level of adiponectin was significantly lower in GDM than CON. The levels of AGEs, and IL-6 were positively correlated with MDA in GDM and CON, and there was a significant negative correlation between adiponectin and MDA in the two groups.
Piuri, G., 2020 [61]	Italy	Cohort study	GDM: 30 CON non pregnant: 53	IADPSG criteria	GDM: 34.0 (32.7-38.5) CON: 36.2	GDM: 23.3 (21.0-26.3)	GDM: 26.0±6	Plasma at diagnosis and after 12 weeks of strict dietetic therapy.	MGO and GA measured by ELISA	BAFF, TNF-α, and PAF measured by ELISA	PAF and TNF-α levels increased in GDM after diet. MGO levels were significantly higher in women with GDM, both at diagnosis and after 12 weeks compared with CON. Levels of GA were significantly

					(31.1-39.3)						higher in women with GDM after 12 weeks of diagnosis compared with general population.<br>PAF levels at diagnosis and after 12 weeks were positively correlated with HbA1c levels and HOMA-IR. MGO levels were positively correlated with HbA1c both at diagnosis and after 12 weeks. MGO was significantly correlated with HOMA-IR at diagnosis.
											MGO levels were positively correlated with both the prepregnancy weight, at GDM diagnosis and after 12 weeks and with the birth weight. GA levels at GDM diagnosis were positively correlated with prepregnancy weight and BMI.
Liu, X., 2020 [62]	China	Case–control study	GDM: 22<br>CON: 22	OGTT during 24-28 weeks. Subjects were considered to have GDM according to two criteria: first, fasting morning venous plasma glucose reached or exceeded 5.1 mmol/L; second, venous plasma glucose levels reached or exceeded two or more of the following values: a fasting morning plasma glucose of 5.1 mmol/L; a 1 hour post-load glucose of 10.0 mmol/L; a 2 hour post-load glucose of 8.5 mmol/L.	GDM: 30.7±2.6<br>CON: 29.2±2.1	GDM: 24.1±4.7<br>CON: 20.8±1.2	GDM: 38.4±1.7<br>CON: 38.9±1.2	Plasma	Protein expression levels of oxidative stress proteins measured by liquid chromatography-tandem mass spectrometry (LC–MS/MS) analysis.	Levels of inflammatory protein expression measured by LC–MS/MS and CRP measured by ELISA to validate the data.	CRP was induced by GDM, and ELISA analysis verified the abnormal expression of CRP in the first-trimester maternal plasma in women who subsequently developed GDM.<br>Global correlation analysis indicated existence of protein coregulations, among 52 inflammation system proteins, and 4 antioxidative stress proteins (extracellular superoxide dismutase, peroxiredoxin-1, serum haptoglobin, and paraoxonase/arylesterase 1).<br>The coregulations of inflammation, oxidative stress, insulin resistance, blood coagulation, and lipid homeostasis were also revealed in GDM development.
Kopylov, A.T., 2020 [63]	Russia	Prospective case–control study	GDM: 110<br>CON: 30	IADPSG criteria	GDM: 26.2±5.3<br>CON: 26.6±5.2	GDM: 25.1±4.8<br>CON: 22.7±3.3	GDM: 23–28<br>CON: 23–28	Plasma	Protein expression levels of oxidative stress proteins measured by liquid chromatography-tandem mass spectrometry (LC–MS/MS) analysis.	Levels of inflammatory protein expression measured by LC–MS/MS	One of the molecular processes involved in the progression of GDM was the stimulation of glycation and ROS generation on NF-kb signaling and TNF- α and IL-6 production

BMI: body mass index, GA: gestational age, GDM: gestational diabetes mellitus, CON: control group ,X: xanthine, XO: xanthine oxidase, ELISA: enzyme-linked immunosorbent assay, EMSA: electrophoretic mobility shift assay, IL: Interleukin, TNF-α: Tumor necrosis factor-alpha, NFκB: nuclear factor-kappa B, EMSA:, LPS: lipopolysaccharide, HX: hypoxanthine, CAT: Catalase, GSR: glutathione reductase, GPX: glutathione peroxidase, SOD: superoxide dismutase, RT–PCR: reverse transcription polymerase chain reaction, G-CSF: granulocyte-colony stimulating factor, GM-CSF: Granulocyte macrophage colony-stimulating factor, IFNγ: interferon gamma, MCP1: Monocyte chemotactic protein-1, MIP1β: Macrophage inflammatory protein-1 beta, IADPSG: International Association of Diabetes and Pregnancy Study Groups, OGTT: Oral glucose tolerance test, TOS: Total oxidant status, TAS: total antioxidant status, PON: paraoxonase, SPON: stimulated paraoxonase SPON, ARES: arylesterase, OSI: Oxidative stress index, CER: Ceruloplasmin, Trf: Transferrin, NT: nitrotyrosine, hs-CRP: High-sensitivity C reactive protein, GTX: Glutathione Peroxidase, MDA: Malondialdehyde, AGEs: Advanced glycation end products, MGO: Methylglyoxal, GA: glycated albumin, BAFF: B-cell activating factor, PAF: platelet-activating factor, HbA1c: glycated hemoglobin, HOMA-IR: Homeostatic Model Assessment of Insulin Resistance , LC–MS/MS: liquid chromatography-tandem mass spectrometry, CRP: C-reactive protein.

antioxidants

MDPI

Supplementary Table S2. Effect of supplements on oxidative stress and inflammation in women with GDM and in cell models of GDM.

Author, Year [Reference]	Study desgin	Sample size	Age	Intervention	Sample	Oxidative stress biomarker	Inflammatory biomarker	Main findings
Hajifaraji, M., 2018 [70]	Double blind placebo controlled randomized clinical trial	64 newly diagnosed GDM: 29 intervention, 27 placebo	27.3±5.8	Probiotic supplements: Lactobacillus acidophilus LA-5, Bifidobacterium BB-12, Streptococcus Thermophilus STY-31 and Lactobacillus delbrueckii bulgaricus LBY-27 or placebo capsule for 8 consecutive weeks	Serum	MDA, GSHR, GPx, SOD	hsCRP, TNF-α, IL-6	Serum hsCRP, and TNF-α levels improved in the probiotic group over the placebo group. Serum IL-6 levels decreased in both groups after intervention; however, neither within group nor betweengroup differences in IL-6 serum levels were statistically significant. MDA, GSHR, and GPx levels improved significantly with the use of probiotics when compared with the placebo
Jamilian, M., 2019 [72]	Randomized, double-blind, placebo-controlled trial	60 women with GDM assigned to receive either 100 mg magnesium, 4 mg zinc, 400 mg calcium plus 200 IU vitamin D supplements (n = 30) or placebo (n = 30)	Intervention group: 27.7±4.0 Placebo group: 29.1±4.1	Magnesium-zinc-calcium-vitamin D supplements	Serum and plasma	MDA, TAC, and GSH	hs-CRP	Magnesium-zinc-calcium-vitamin D cosupplementation resulted in a significant reduction in serum hs-CRP and plasma MDA concentrations, as well as a significant increase in TAC levels, compared to placebo.
				**In vitro model of GDM**				
Nguyen-Ngo C., 2019 [75]	In vitro model of GDM	16 NG women at term elective Cesarean section	NR	Naringenin	Placenta, VAT and SAT, skeletal muscle explants stimulated with TNF-α	SOD, catalase, and GSHR mRNA expression	IL1B, IL6, CCL2, CXCL1, CXCL8 mRNA expression and protein release. NF-κB Pathway	Naringenin treatment significantly improved TNF-impaired glucose uptake in skeletal muscle. In placenta VAT, naringenin significantly reduced expression of pro-inflammatory cytokines and chemokines and increased antioxidant mRNA expression.
Nguyen-Ngo C., 2020 [76]	In vitro model of GDM	27 NG nonobese women at term elective Cesarean section	NR	Punicalagin, curcumin	Placenta, VAT and SAT explants stimulated with TNF-α	SOD, catalase mRNA expression	TNF-α, IL-1A, IL-1B, IL-6, CCL2-4, CXCL1, CXCL5, CXCL8 mRNA expression	Punicalagin and curcumin significantly suppressed TNF- α induced pro-inflammatory cytokine (IL1A, IL1B, and IL6) and chemokine (CCL2-4, CXCL1, CXCL5 and CXCL8) expression in human placenta, VAT and SAT. Anti-inflammatory cytokine IL4 and IL13 mRNA expression was also upregulated by punicalagin and curcumin treatment in placenta, VAT and SAT. Punicalagin and curcumin also altered antioxidant (SOD and catalase) mRNA expression in placenta, VAT and SAT.
Nguyen-Ngo C., 2022 [79]	In vitro model of GDM	6 NG women with a BMI < 30 kg/m² who were delivering healthy, singleton infants at term (37–41 weeks of gestation) via elective Cesarean section	NR	Selenium	Placental tissue, VAT and SAT stimulated with LPS and TNF-α	GPx and TrxR	GM-CSF, IL1A, IL1B, IL6, CCL2, CCL4, CXCL1, CXCL5 and CXCL8	Selenium pretreatment blocked LPS and TNF-α induced mRNA expression and secretion of pro-inflammatory cytokines and chemokines, while increasing anti-inflammatory cytokine and antioxidant mRNA expression in placenta, VAT and SAT. Selenium pretreatment was also found to inhibit LPS- and TNF-α induced phosphorylation of ERK in placenta, VAT and SAT.

GDM: gestational diabetes mellitus, MDA: malondialdehyde, GSHR: glutathione reductase, GPx: glutathione peroxidase, SOD: superoxide dismutase, hs-CRP: high-sensitivity C reactive protein, TNF-α: tumor necrosis factor-alpha, IL: interleukin, TAC: total antioxidant capacity, GSH: glutathione, NGT: normal glucose tolerant, NG: normoglycemic, NR: not reported, VAT: visceral adipose tissue, SAT: subcutaneous adipose tissue, CCL2: monocyte chemoattractant protein-1, CXCL1: C-X-C motif chemokine ligand 1, CXCL8: C-X-C motif chemokine ligand 8, NFκB: nuclear factor-kappa B, CXCL5: C-X-C motif chemokine ligand 5, LPS: lipopolysaccharide, TrxR: thioredoxin reductase, GM-CSF: granulocyte macrophage colony-stimulating factor, CCL4: C-C motif chemokine ligand 4, ERK: extracellular signal-regulated kinase.
